# Spatiotemporal Regulation of CRISPR/Cas9 Enables Efficient, Precise, and Heritable Edits in Plant Genomes

**DOI:** 10.3389/fgeed.2022.870108

**Published:** 2022-04-26

**Authors:** Farhanur Rahman, Apurva Mishra, Archit Gupta, Rita Sharma

**Affiliations:** ^1^ Department of Biological Sciences, Birla Institute of Technology and Science (BITS), Pilani, India; ^2^ Department of Molecular and Cellular Biology, University of California, Davis, Davis, CA, United States

**Keywords:** Cas9, inducible promoter, tissue-specific promoter, genome editing, CRISPR

## Abstract

CRISPR/Cas-mediated editing has revolutionized crop engineering. Due to the broad scope and potential of this technology, many studies have been carried out in the past decade towards optimizing genome editing constructs. Clearly, the choice of the promoter used to drive gRNA and Cas9 expression is critical to achieving high editing efficiency, precision, and heritability. While some important considerations for choosing a promoter include the number and nature of targets, host organism, mode of transformation and goal of the experiment, spatiotemporal regulation of Cas9 expression using tissue-specific or inducible promoters enables higher heritability and efficiency of targeted mutagenesis with reduced off-target effects. In this review, we discuss specific studies that highlight the prospects and trade-offs associated with the choice of promoters on genome editing and emphasize the need for inductive exploration and discovery to further advance this area of research in crop plants.

## 1 Introduction

The Clustered, Regularly Interspaced, Short Palindromic Repeats (CRISPR)/CRISPR-associated protein (Cas) system is a powerful system for programmable editing of gene structure, expression, and epigenetics (Jinek et al., 2012; Adli, 2018). Unlike other genome editing tools such as meganucleases (MGN), zinc finger nucleases (ZFNs), and transcription activator-like effector-based nucleases (TALENs), the level of precision, flexibility, and ease of application and design of the CRISPR/Cas system is unparalleled (Huang et al., 2015; Arora and Narula, 2017). Further, due to its ability to target multiple genes, the CRISPR/Cas system can address the challenge of high redundancy in plant genes while establishing gene-function relationships (Xing et al., 2014; Abdelrahman et al., 2021).

The major components of the CRISPR/Cas9-mediated genome editing toolkit are Cas9 endonuclease and guide RNA (gRNA), which form a complex and cleave the target DNA adjacent to the protospacer adjacent motif (PAM). The gRNA acts as a guide for the Cas9 protein to make the double-strand breaks (DSBs) at the target site, which may either be repaired through an error-prone non-homologous end-joining (NHEJ) or the high-fidelity homologous recombination (HR) pathway (Iliakis et al., 2004; Wyman and Kanaar, 2006; Heyer et al., 2010). The optimization of the construct design, especially codon optimization of Cas9, gRNA design, and regulatory regions (promoters) used for driving gRNA and Cas9 expression, are critical to achieving high precision, targeting efficiency and heritability (Li et al., 2013; Johnson et al., 2015; Hassan et al., 2021). The efficiency is usually reported as the percentage of transgenic plants with a mutation at the intended target, while precision is assessed based on the frequency of off-target mutations. Heritability is estimated based on the number of plants that inherit the targeted mutation from one generation to another. Here, we highlight the impact of spatiotemporal regulation of Cas9/gRNA expression on mutagenesis efficiency, precision, and heritability of genome edits in plants.

### 1.1 Different Architectures of Clustered, Regularly Interspaced, Short Palindromic Repeats Constructs

**FIGURE 1:**
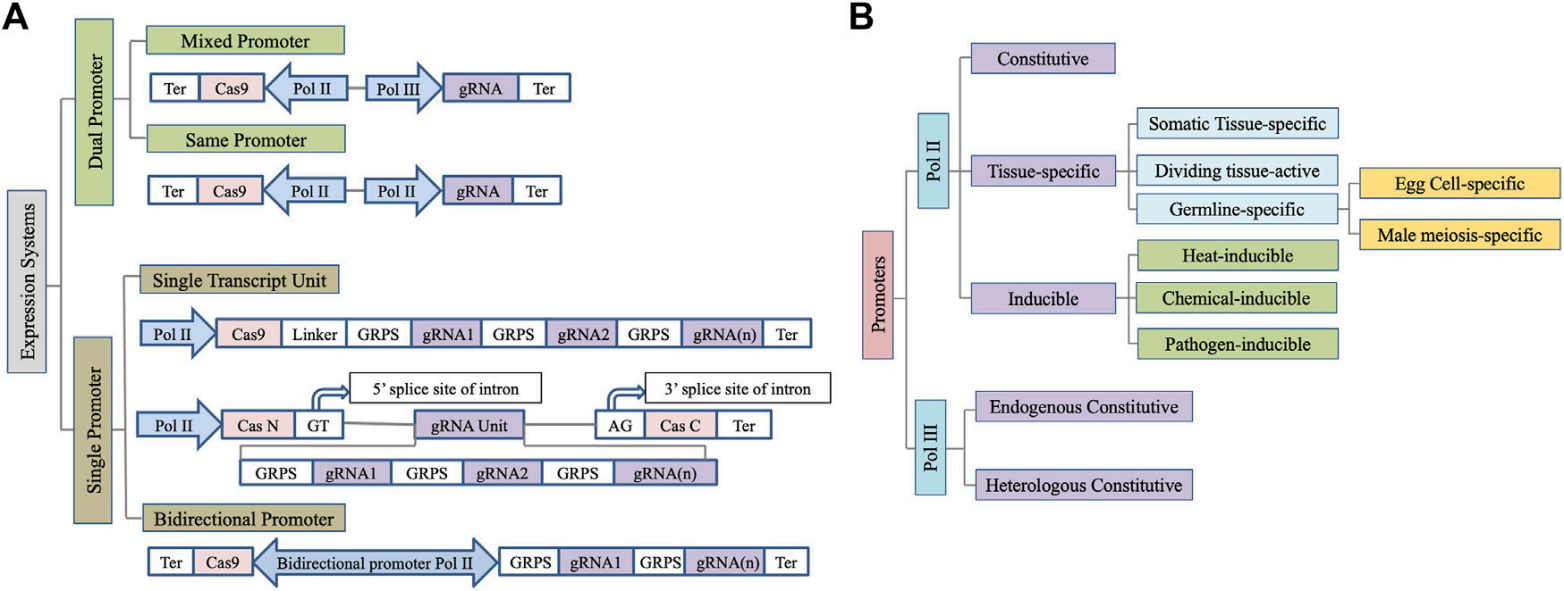
Schematic depiction of wide range of **(A)** expression systems and **(B)** promoters used for Cas9/gRNA expression in plants. Ter, Terminator; GRPS, Guide RNA processing system.

Several architectures of the expression cassette for Cas9 and gRNA expression are being used in plants (Figure 1A) (Montecillo et al., 2020). A mixed dual promoter system has most frequently been used where a Pol II promoter drives Cas9 expression while a Pol III promoter is used for gRNA expression (Lowder et al., 2015). The use of two distinct Pol II promoters for Cas9 and sgRNA expression has also been successfully demonstrated (Čermák et al., 2017). Pol II promoters are especially useful for transcribing multiple gRNAs as a single transcriptional unit to facilitate multiplex editing when coupled with either self-cleaving hammerhead (HH) and hepatitis delta virus (HDV) ribozymes, bacterial CRISPR-associated RNA endoribonuclease Csy4, or endogenous tRNA processing enzymes to facilitate gRNA processing (Čermák et al., 2017). Since increasing the construct size by using two distinct Pol II promoters for Cas9 and sgRNA expression puts an additional constraint on transformation in plants, a single transcript unit (STU) system where a single promoter is used to express both sgRNAs and Cas9 is also effective (Tang et al., 2019). Insertion of sgRNA in the intron of the Cas9 further enhances the system’s efficiency (Figure 1A) (Ding et al., 2018). However, repeated use of the same Pol II promoter to express the gRNA and Cas9 risks homology-dependent gene silencing (Rajeevkumar et al., 2015). Recently, the use of bidirectional promoters to regulate Cas9 and gRNA expression has also been successfully demonstrated in plants (Ren et al., 2019). It facilitates coordinated expression of Cas9 and gRNA while shortened vector would be advantageous during plant transformation. However, due to a limited number of bidirectional promoters characterized to date, this system needs further investigations for wider applicability. Below we encapsulate the wide range of promoters that have been leveraged for genome editing in plants so far (Figure 1B).

### 1.2 Constitutive Promoters

Constitutive promoters with sustained high expression in all cell types have been extensively used in transgenic systems. Both constitutive RNA Polymerase II (Pol II) and III (Pol III) promoters have been leveraged for genome editing applications in plants.

#### 1.2.1 Polymerase II Constitutive Promoters

Constitutive Pol II promoters derived from plant pathogens (e.g., CaMV, NOS) or housekeeping genes (e.g., *Ubiquitin*, *Actin, EF1A2*) have been most widely used for driving Cas9 expression in plants with endogenous promoters exhibiting higher efficiency over heterologous promoters (Li et al., 2015). Besides, *Cauliflower mosaic virus* (CaMV) 35S promoter exhibits greater efficiency in dicots while maize *Ubiquitin* promoter (pZmUbi) exhibits higher mutagenesis rate in monocots (Feng et al., 2013). However, Cas9 driven by constitutive promoter generally leads to the formation of chimeras requiring screening of a large number of edited lines for several generations to obtain homozygous mutants (Feng et al., 2014). Especially in *Arabidopsis,* which is transformed using the floral dip method, constitutive promoters result in mosaics in T1 generation as mutations mainly occur after the first embryonic cell division (Fauser et al., 2014; Feng et al., 2014). Also, despite the high mutagenesis efficiency reported with these promoters, the heritability of mutations remains low due to the limited activity of constitutive promoters in germline cells. Similar results have been reported in soybean, where plant transformation depends on organogenesis and regeneration. Most edits are somatic and non-transmissible in soybean when Cas9 is expressed using a constitutive promoter (Zheng et al., 2020).

#### 1.2.2 Polymerase III Constitutive Promoters

Due to high transcriptional efficiency with short transcripts, Pol III promoters have been extensively used for regulating gRNA expression. As is the case for Pol II promoters, endogenous Pol III promoters exhibit higher editing efficiency (Sun et al., 2015; Long et al., 2018; Qi et al., 2018; Ren et al., 2021). Due to limited characterization of Pol III promoters in less-studied systems, *U6* and *U3* promoters of *Arabidopsis* and rice have been most extensively used for gRNA expression in dicots and monocots, respectively (Ma et al., 2015; Lowder et al., 2016; Montecillo et al., 2020). Trimmed U3 and U6 promoters, with only essential elements for transcriptional initiation, have been used to further shorten the construct (Hao et al., 2020). However, there are some obvious limitations associated with Pol III promoters. For example, earlier U6 and U3 promoters were believed to have a highly conserved transcription start site at +1. Instead, the transcription start site of the U6 promoter seems to vary, which can have undesirable consequences for gRNA specificity (Ma et al., 2014). Some of the studies have also demonstrated Pol II activity of Pol III promoters with varying strength which can have adverse implications on localization and stability of gRNAs (Gao et al., 2018). The dual property (Pol II and Pol III) of the H1 promoter of humans has recently been utilized to drive both guide RNA and Cas9 expression (Gao et al., 2019). Further, Pol III promoters obviously lack spatiotemporal control and tunability, and due to their limited ability to regulate longer transcripts, they are not suitable for multiplex editing applications.

### 1.3 Tissue-Specific Promoters

Since sustained expression of Cas9 using constitutive promoter provides a wider opportunity for off-target effects, spatiotemporal control of Cas9 expression using tissue-specific promoters would be ideal for restricting undesirable Cas9 expression. Based on the experiment’s goal, researchers have employed several germline-specific, cell division-active, and somatic tissue-specific promoters for genome editing in plants (Table 1). We refer to promoters with preferential high expression in meristematic and germline cells as cell division-active as they may have some activity in other cells as well.

**TABLE 1:** List of spatiotemporally regulated promoters used for driving Cas9/gRNA expression in plants.

S. No	Characteristic feature of the promoter	Name of the gene whose promoter was used	Host plant	Remarks	References
**Tissue-specific promoters**					
1	Dividing cell-active	*YAO*	*Arabidopsis*	*YAO* promoter led to higher mutation efficiency in T1 plants compared to constitutive 35S promoter	Yan et al. (2015)
2	Dividing cell-active	*ICU*	*Arabidopsis*	Homozygous mutants recovered in T2 and T3 generation with high heritability	Hyun et al. (2015)
3	Dividing cell-active	*EFα1* and *H4*	*Arabidopsis*	Higher heritability of biallelic mutations in T1 and T2 plants compared to 35S promoter	Osakabe et al. (2016)
4	Dividing cell-active	*RPS5A*	*Arabidopsis*	Efficient editing with high heritability; Biallelic null mutations observed in T1 generation itself with 100% heritability in T2 plants for some genes	Tsutsui and Higashiyama, (2017)
5	Dividing cell-active	*YAO* and *CDC45*	*Arabidopsis*	Multiplex editing using *YAO* and *CDC45* promoters led to higher editing efficiency, heritability and frequency of homozygous mutants compared to 35S promoter in T1 generation	Feng et al. (2018b)
6	Dividing cell-active	*Pcubi4*, *Slp16* and *SIEF1α*	Tomato	Highest mutation efficiency observed with *SIEFα* with reduced number of mosaic mutations in T0 plants	Hashimoto et al. (2018)
7	Meiosis-specific	*MGE1*, *2* and *3*	*Arabidopsis*	MGE1p demonstrated the highest mutation efficiency and heritability out of the three meiosis-specific promoters tested	Eid et al. (2016)
8	Meiosis-specific	*DMC1*	Maize	Homozygous mutants obtained in T0 generation stably inherited to T1 plants with no off-target mutations in the predicted sites	Feng et al. (2018a)
9	Meiosis-specific	*AtDMC1*	*Arabidopsis*	More than 60% T1 plants were heterozygous while 37% of T2 plants were homozygous for the targeted mutation	Xu et al. (2018)
10	Egg cell-specific	*EC1.1*, *EC1.2*	*Arabidopsis*	Fusion promoter developed using the *EC1.1* promoter and *EC1.2* enhancer exhibited higher mutation efficiency	Wang et al. (2015)
11	Egg cell-specific	*AtP5*, *AtEC1.2e1.1*, *GmEC1.1* and *GmEC1.2*	*Arabidopsis* and Soybean	The fusion promoter *AtEC1.2e1.1* led to highest mutation frequency in *Arabidopsis* T2 and soybean T0 plants	Zheng et al. (2020)
12	Male and Female Germline-specific	*SPL*, *DD45* (*EC1.2*) and *LAT52*	*Arabidopsis*	While other germ-line specific promoter led to high heritable heterozygous mutations in T2 plants, only *DD45* promoter generated heritable homozygous mutations in the T1 plants	Mao et al. (2016)
13	Egg cell-specific and dividing cell-active	DD45 (*EC1.2*), LAT52, YAO and CDC45	*Arabidopsis*	*DD45* promoter led to heritable and efficient gene targeting in T2 and T3 plants through HDR	Miki et al. (2018)
14	Egg cell-specific and dividing cell-active	CLV3, YAO and EC1.1	*Arabidopsis*	The egg cell-specific fusion promoter showed highest mutation frequency with heritable events detected in 74% of T1 lines	Wolter et al. (2018)
15	Egg cell-specific and dividing cell-active	*RPS5a*, *DD45* (*EC1.2*), *35S*, *PcUbi*, *AP1*, *ICU2*, *GILT* and *ALB*	*Arabidopsis*	Promoters of *RPS5a* and *DD45* showed superior performance among all the tested promoters	Ordon et al. (2020)
16	Fibre-specific	*NSD3*/*SND1*	*Arabidopsis*	Cell-specific editing of *HCT* achieved and maintained in T1 and T2 generation	Liang et al. (2019)
17	Fruit-specific	*PPC2*	Tomato	Target gene editing was achieved in fruits with some leakiness in seeds	Feder et al. (2020)
18	Root cap-specific	*SMB*	*Arabidopsis*	Simultaneous editing of six genes achieved with high editing efficiency in T1 and T2 plants	Bollier et al. (2021)
**Inducible Promoters**					
19	Estrogen-inducible	G10-90	Rice Protoplasts	Single transcript unit system with Cas9, sgRNA and ribozyme expressed using single estradiol-inducible promoter for editing	Tang et al. (2019)
20	Estrogen-inducible	*WOL*, *WOX5*, *SCR* and *WER*	*Arabidopsis*	Root cell-specific inducible editing achieved	Wang et al. (2020)
21	Heat-inducible	Soybean *HSP17.5E*	Rice	Higher mutation frequencies with significantly reduced off-target effects compared to constitutive rice ubiquitin promoter in T0 plants	Nandy et al. (2019)
22	Heat-inducible	*Hsp26*	Maize	High-frequency intragenomic HR led to non-chimeric heritable gene targeting in T0 plants	Barone et al. (2020)
23	Heat-inducible	*Arabidopsis Heat shock promoter 18.2*	Tobacco BY2 cells	Heat-inducible expression of sgRNA used to excise the target T-DNA boundaries for excision of the transgene after successful editing	Hanania et al. (2020)
24	Pathogen (BSCTV)-inducible	*V86* and *C86*	*Arabidopsis* and Tobacco (transient)	No off target effects observed in T2 *Arabidopsis* plants with virus-inducible promoters	Ji et al. (2018)

#### 1.3.1 Cell Division-Active Promoters

Promoters of several genes active in proliferating cells of shoot apical meristem, root meristem, young leaves, anthers, pollen, embryo sac, embryo, and endosperm such as *YAO* (*YAOZHE*), *ICU* (*INCURVATA*), *EFα1* (*Elongation factor alpha 1*), *H4* (*Histone 4*), *RPS5A* (*Ribosomal Protein S5A*), *CDC45* (*Cell division control protein 45*) have been leveraged for driving Cas9 expression in *Arabidopsis*. All these studies report higher editing efficiency and heritability of homozygous mutations with dividing tissue-specific promoters than constitutive promoters (Hyun et al., 2015; Yan et al., 2015; Osakabe et al., 2016; Tsutsui and Higashiyama, 2017; Feng Z. et al., 2018). Some of the genes even exhibited homozygous mutations in T1 generation itself with 100% heritability in T2 plants with *RPS5A* promoter-driven Cas9 (Tsutsui and Higashiyama, 2017). Later, Feng and co-workers (2018b) demonstrated higher editing efficiency, heritability, and frequency of homozygous mutants in T1 plants of *Arabidopsis* for multiplex editing by using *YAO* and *CDC45* promoters compared to 35S promoter. Similar results were reported in tomato as well where three constitutive promoters, including *CaMV35S*, *Pcubi4* (*Polyubiquitin 4*), and *Slp16* (*Ribosomal protein p16*) were compared with the promoter of *Elongation Factor-1alpha* (*SIEF1α*) which is primarily active in meristematic cells of shoot tips and shoot apical meristems. The authors observed the highest mutation efficiency with *SIEFα* with the reduced number of mosaic mutations in T0 plants (Hashimoto et al., 2018). These results demonstrate the superiority of dividing cell-active promoters over constitutive promoters for individual loci and multiplex editing in plants.

#### 1.3.2 Germlines-Specific Promoters

With Cas9 expression restricted to germline cells, male meiosis and egg cell-specific promoters have the potential to enhance the heritability of mutations and reduce the frequency of somatic mutations. Both male and female germlines-specific promoters have been leveraged for this purpose.

##### 1.3.2.1 Male Meiosis-Specific Promoters

Eid and co-workers (2016) tested three meiosis-specific promoters for driving Cas9 expression in *Arabidopsis*. *MGE1p* is specific to meiosis I, *MGE2p* is specific to meiosis I and II, while *MGE3p* is specific to meiosis II. All three promoters mediated mutagenesis in T1 plants, with *MGE1p* exhibiting the highest efficiency, while *MGE3p* had the lowest efficiency. *MGE1p* even outperformed the egg-cell-specific promoter of *EC1.2p*. Similarly, Xu and co-workers (2018) used meiocyte-specific promoter *DMC1* (*DNA Meiotic Recombinase 1*), which is active in both male and female meiocytes, to drive Cas9 expression in *Arabidopsis* and reported 64% T1 plants as heterozygous while 37% of the T2 plants were homozygous for the targeted mutation. Conversely, the use of the *DMC1* promoter for regulating Cas9 expression in maize led to 66% homozygous mutants in T0 generation itself which were stably inherited in T1 plants (Feng C. et al., 2018). Moreover, no off-target mutations were detected in the predicted loci. Since transgenic plants were regenerated using tissue culture procedure in maize, high expression of *DMC1* in calli as well as germline cells likely contributed to the high mutagenesis efficiency with this promoter which can be leveraged for other grain crops as well.

##### 1.3.2.2 Egg Cell-Specific Promoters

Cas9 expression driven by *EC1.2* (*Egg Cell 1.2*)*/DD45/*(*Downregulated in dif1 45*) promoter that exhibits specific expression in the egg cells and one-cell stage embryos led to homozygous mutants in T1 *Arabidopsis* plants (Wang et al., 2015). Although its molecular basis is still unclear, a fusion promoter developed using the *EC1.1* promoter and *EC1.2* enhancer further improved the mutation efficiency. A similar observation was made in another study with *Arabidopsis* and soybean where four different promoters, including egg cell-specific promoters, *AtP5p* and *AtEC1.2e1.1* of *Arabidopsis*, and *GmEC1.1p* and *GmEC1.2p* of soybean, were utilised for Cas9 expression (Zheng et al., 2020). The *AtEC1.2e1.1* promoter resulting from the fusion of *cis*-regulatory elements of the *EC1.1* and *EC1.2* promoters exhibited higher mutation frequency than the other three promoters in T2 plants of *Arabidopsis*. In soybean, *AtEC1.2e1.1* led to a mutation rate of 26.8%, while no mutations could be detected with the other three promoters in T0 soybean plants. Although the number of transgenic soybean lines was too low to draw a clear conclusion, both the studies clearly suggest that an optimized combination of egg cell-specific promoters can enhance the mutation efficiency and proportion of homozygous mutations.

Mao and co-workers (2016) compared the efficiency of *DD45* promoter with male germline-specific promoters, *SPL* (*SPOROCYTELESS*) and *LAT52* in *Arabidopsis* (Mao et al., 2016)*. SPL* predominantly expresses in early microsporocytes while *LAT52* expresses during late pollen development. Though all germ-line specific promoters led to heritable heterozygous mutations in the T2 generation, only *DD45* promoter led to heritable homozygous mutations in the T1 generation. The authors speculate that since the primary target in the floral dip transformation method in *Arabidopsis* is ovules, the expression of *DD45* promoter in zygotes might be synchronized with *Agrobacterium* infection of egg cells resulting in homozygotes in the T1 generation. Later, Miki and co-workers (2018) compared the *DD45* and *LAT52* promoters with dividing tissue-active *YAO* and *CDC45* promoters for highly efficient RNA-targeted gene knock-in (targeted insertions of external genes) in *Arabidopsis*. The authors developed “all-in-one” constructs with HDR (Homology-directed Repair) donor sequence and sgRNA targeting genomic locus of interest for *GFP* knock-in into the *ROS1* and *DME* (*DEMETER*) gene loci. Yet again, while precise knock-ins that could produce *ROS1-GFP*, *DME-GFP*, or *GFP-DME* fusions were accomplished with other promoters, only *DD45* promoter led to heritable and efficient gene targeting in T2 and T3 plants, indicating that HDR may be more efficient in egg cells and/or early embryos. Wolter and co-workers (2018) compared the mutation efficiency of promoters of stem-cell identity regulator *CLV3* (*CLAVATA3*) with *YAO* and *EC1.1* for gene targeting by homologous recombination (HR) in *Arabidopsis*. While *CLV3* and *YAO* promoters exhibited very low GT frequency like constitutive ubiquitin promoter, the egg cell-specific fusion promoter *EC1.1* exhibited high genome targeting frequency with heritable events in 74% of the T1 lines (Wolter et al., 2018). Comparative analysis of editing efficiencies of *RPS5a* and *DD45* promoters with several constitutive and tissue-specific promoters including *p35S*, *pPcUbi*, *pAP1*, *pICU2*, *pGILT*, and *pALB* to target *Lhcb1* genes, which is present in five copies at two loci in the *Arabidopsis* genome also confirmed the superior performance of *pRPS5a* and *DD45* among all the tested promoters (Ordon et al., 2020). These studies demonstrate the application of egg cell-specific promoters for wide range of genome editing applications.

#### 1.3.3 Somatic Tissues-Specific Promoters

In situations where gene editing can be detrimental to the organism’s survival or lead to pleiotropic effects, hindering the further experimental investigation, somatic tissue-specific editing can be performed. For instance, to analyze the function of *Hydroxycinnamoyl transferase* (*HCT*), which is required for lignin biosynthesis in *Arabidopsis*, Liang and co-workers (2019) used a fibre-specific promoter *pNSD3*/*SND1* to drive Cas9 expression. With a 90% decrease in *HCT* activity, the chimera plants exhibited a normal growth phenotype allowing biochemical analysis of the plants while the cell-specific editing was maintained in the T2 generation. Another fascinating example of using tissue-specific promoters for editing essential genes comes from a study done by Feder and co-workers (2020), where the authors demonstrated the use of fruit-specific *phosphoenolpyruvate carboxylase 2* (*PPC2*) promoter in tomato for editing the SET-domain containing polycomb gene, SIEZ2, which earlier yielded pleiotropic phenotypes when targeted using RNA interference (Boureau et al., 2016). Although *PPC2* promoter exhibited leakiness in seeds, the study allowed investigation of additional roles of *SIEZ2* in fruit maturation. More recently, Bollier and co-workers (2021) used this approach for multigene editing of fundamentally important genes using root cap-specific promoter *pSMB* (*SOMBRERO*). The authors confirmed the editing of six genes simultaneously with high efficiency in T1 and T2 *Arabidopsis* plants. This system allows the knockdown of multiple genes with redundant or synergistic functions to dissect cell/tissue-specific genetic networks while maintaining the sequence integrity in germline cells.

### 1.4 Inducible Promoters

While germline-specific promoters help deal with the challenge of lower heritability of mutations, the inducible promoters may further reduce the off-target effects due to transient expression of Cas9. The inducible systems respond to either chemical inducers or biotic/abiotic stimuli (Qiu and Yu, 2009; Misra and Ganesan, 2021).

#### 1.4.1 Chemical-Inducible Promoters

Several chemicals such as estradiol, ethanol, ecdysone, glucocorticoid, etc., have been used for developing inducible systems in plants. However, some of these can lead to untended growth defects (Kang et al., 1999; Roslan et al., 2001; Amirsadeghi et al., 2007). The estrogen-inducible chimeric transcription activator (XVE) system has been successfully used in several plant species without any undesirable impact on plant growth and morphology. Tang and workers (2019) used the XVE system to demonstrate the application of an STU system where Cas9, sgRNA, and ribozyme were expressed using a single estradiol-inducible promoter for regulated expression of Cas9 and sgRNA in the rice protoplast system. The authors observed leaky expression of Cas9 in some samples with low level of mutagenesis detected without estradiol treatment.

Recently Wang and co-workers (2020) demonstrated the application of an inducible editing system for creating gene knockouts in *Arabidopsis*. The authors tested four inducible promoters viz., *pWOL*: *XVE*, *pWOX5*:*XVE*, *pSCR*: *XVE*, and *pWER*: *XVE*, which drive expression in specific cell types of root meristem to target the *PLT2* (*PLETHORA2*) gene. The *PLT2* editing was assessed using YFP fluorescence as an indicator. Further, the cell-type specificity of their system in root meristems was demonstrated by targeting AP2/EREBP family transcription factor genes, *PLT1* and *PLT2*. The double mutant *plt1,2* phenotype was rescued using gPLT2-3xYFP, where 3xYFP was used to restrict the mobility of *PLT2*, thereby allowing tracking of cell-specific editing of *PLT2*. Results suggest that the cell-type-specific inducible system is a promising strategy for generating cell-type-specific high-precision gene knockouts.

#### 1.4.2 Heat-Inducible Promoters

A heat-inducible promoter is induced by a high-temperature stimulus. There have been three studies so far demonstrating heat-inducible promoters for editing applications. Nandy and co-workers (2019) used promoter of soybean *heat shock protein 17.5E* (*HSP17.5E*) for driving Cas9 expression for targeted editing of endogenous *phytoene desaturase* (*PDS*) gene and *β-Glucuronidase* transgene in rice. The heat-shock treatment of calli/seedlings led to heritable heterozygous and homozygous mutations in T0 plants with more than 3 times mutation frequency and significantly reduced off-target effects compared to constitutive rice ubiquitin promoter. Conversely, Barone and co-workers (2020) demonstrated the use of heat shock inducible promoter *Hsp26* to facilitate intragenomic gene targeting in maize. After transformation, induction of Cas9 in undifferentiated cells led to DSBs with simultaneous mobilization of donor template from pre-integrated T-DNA. High-frequency intragenomic HR led to non-chimeric heritable gene targeting with 4.7% target insertion in T0 plants. More recently, Hanania and colleagues (2020) used *18.2 Arabidopsis* heat shock promoter to drive sgRNA expression, specially designed to excise the target T-DNA boundaries for excision of the transgene from *Nicotiana tabacum* BY2 (Bright Yellow 2) cells after confirmation of successful editing. Together these studies demonstrate wide applications of heat-inducible promoters in plants provided the duration and cycles of heat treatment are optimised.

#### 1.4.3 Pathogen-Inducible Promoters

Pathogen-inducible promoters are a promising strategy to engineer disease resistance in crop plants. Ji and co-workers (2018) demonstrated the use of a virus-inducible CRISPR/Cas system to engineer resistance against beet curly top virus (BSCTV) in tobacco transient and *Arabidopsis* transgenic systems. After observing off-target effects in T2 plants using constitutive promoters, the authors tested BSCTV-inducible *pV86* and *pC86* promoters for Cas9 expression. Since the pathogen itself induces pathogen inducible systems, these are ideal for engineering disease resistance without needing an inducer and worrying about the mode of application. Also, as no other factors have been shown to induce these promoters, no leaky expression and off-target effects were detected in the absence of the pathogen.

## 2 Conclusions and Future Perspectives

It is evident from the above studies that regardless of the system used, spatiotemporal regulation of Cas9 enables greater precision and heritability compared to constitutive promoters. The dividing tissue-active promoters enhance the mutation efficiency, heritability and proportion of homozygous mutants in *Arabidopsis*. The germline-specific promoters further decrease the chimerism by limiting Cas9 expression to specific cell types along with improved heritability and frequency of homozygous mutations. Further, egg cell-specific expression of Cas9 has been shown to stimulate CRISPR-dependent HDR more efficiently (Miki et al., 2018). Somatic tissue-specific promoters, on the other hand, are a boon for characterizing essential genes without disturbing the integrity of germline cells. However, in cases where target tissue types may not have promoters with high enough activity to be deployed for editing, synthetic/fusion promoters may be designed to fine-tune the regulatory behaviours of the known regulatory elements (Ali and Kim, 2019; Zhang et al., 2020).

Inducible promoters provide tighter spatial as well as temporal control and therefore, would be an ideal system for cell type-specific high precision edits for both essential and non-essential genes. Use of transient expression of inducible promoters compared to somatic tissue-specific promoters for characterizing essential genes would likely reduce the frequency of somatic mutations also. However, no studies have been conducted so far in plants to compare the performance of the inducible promoters with tissue-specific promoters. The leaky expression, speed of response and limited mobility of inducer may be of concern especially when the target tissue is deep seated like ovules and embryo sacs. Further research is needed to fully explore the potential of inducible promoters vis-à-vis tissue-specific promoters.

Another major lacuna is that most of the studies described here have been conducted in *Arabidopsis* which has a very different mode of transformation compared to the crop species that are dependent on tissue culture procedures for transgenic generation. Also, variable response of some of these promoters has been observed in different host systems and laboratories (Shockey, 2020). Some of the factors that likely contribute to this variability include variable length of regulatory regions used in different studies, host-specific factors, variability in sequence of gRNAs and target region, and impact of other regulatory elements in the vector used. Therefore, comparative assessment of spatiotemporally-regulated promoters across different plant species would be required to assess the wider applicability of tissue-specific and inducible promoters beyond *Arabidopsis*. Considering the exponential advancements this technology has witnessed in the past decade and its societal impact, the quest for enhanced efficiency, specificity and heritability of Cas9-mediated editing will hopefully lead to a wider assortment of promoters in the future which can then be used for developing recommender systems for specific host species and genome editing applications.
